# Phase II trial of vaccination with autologous, irradiated melanoma cells engineered by adenoviral mediated gene transfer to secrete granulocyte-macrophage colony stimulating factor in patients with stage III and IV melanoma

**DOI:** 10.3389/fonc.2024.1395978

**Published:** 2024-05-15

**Authors:** Tamara A. Sussman, Mariano Severgnini, Anita Giobbie-Hurder, Philip Friedlander, Scott J. Swanson, Michael Jaklitsch, Thomas Clancy, Laura A. Goguen, David Lautz, Richard Swanson, Heather Daley, Jerome Ritz, Glenn Dranoff, F. Stephen Hodi

**Affiliations:** ^1^ Department of Medical Oncology, Dana-Farber Cancer Institute, Boston, MA, United States; ^2^ Department of Clinical Sciences, Curis, Inc., Lexington, MA, United States; ^3^ Division of Biostatistics, Department of Data Science, Dana-Farber Cancer Institute, Boston, MA, United States; ^4^ Department of Hematology and Oncology, Icahn School of Medicine at Mount Sinai, New York, NY, United States; ^5^ Department of Surgery, Brigham and Women’s Hospital, Boston, MA, United States; ^6^ Division of Otolaryngology, Brigham and Women’s Hospital, Boston, MA, United States; ^7^ Department of Surgery, Emerson Hospital, Concord, MA, United States; ^8^ Department of Surgery, UMass Chan Medical School, Worcester, MA, United States; ^9^ Connell and O’Reilly Families Cell Manipulation Core Facility, Dana-Farber Cancer Institute, Boston, MA, United States; ^10^ Department of Medical Oncology, Parker Institute for Cancer Immunotherapy, Dana-Farber Cancer Institute, Boston, MA, United States

**Keywords:** melanoma, vaccine, advanced disease, GM-CSF, stage III, Stage IV

## Abstract

**Background:**

In the era of immune checkpoint blockade, the role of cancer vaccines in immune priming has provided additional potential for therapeutic improvements. Prior studies have demonstrated delayed type hypersensitivity and anti-tumor immunity with vaccines engineered to secrete granulocyte-macrophage colony-stimulating factor (GM-CSF). The safety, efficacy and anti-tumor immunity of GM-CSF secreting vaccine in patients with previously treated stage III or IV melanoma needs further investigation.

**Methods:**

In this phase II trial, excised lymph node metastases were processed to single cells, transduced with an adenoviral vector encoding GM-CSF, irradiated, and cryopreserved. Individual vaccines were composed of 1x10^6^, 4x10^6^, or 1x10^7^ tumor cells, and were injected intradermally and subcutaneously at weekly and biweekly intervals. The primary endpoints were feasibility of producing vaccine in stage III patients and determining the proportion of patients alive at two years in stage IV patients.

**Results:**

GM-CSF vaccine was successfully developed and administered in all 61 patients. Toxicities were restricted to grade 1-2 local skin reactions. The median OS for stage III patients (n = 20) was 71.1 (95% CI, 43.7 to NR) months and 14.9 (95%CI, 12.1 to 39.7) months for stage IV patients. The median PFS in stage III patients was 50.7 (95%CI, 36.3 to NR) months and 4.1 (95% CI, 3.0-6.3) months in stage IV patients. In the overall population, the disease control rate was 39.3% (95%CI, 27.1 to 52.7%). In stage III patients, higher pre-treatment plasma cytokine levels of MMP-1, TRAIL, CXCL-11, CXCL-13 were associated with improved PFS (p<0.05 for all). An increase in post-vaccination levels of IL-15 and TRAIL for stage III patients was associated with improved PFS (p=0.03 for both). Similarly, an increase in post-vaccination IL-16 level for stage IV patients was associated with improved PFS (p=0.02) and clinical benefit.

**Conclusions:**

Vaccination with autologous melanoma cells secreting GM-CSF augments antitumor immunity in stage III and IV patients with melanoma, is safe, and demonstrates disease control. Luminex data suggests that changes in inflammatory cytokines and immune cell infiltration promote tumor antigen presentation and subsequent tumor cell destruction. Additional investigation to administer this vaccine in combination with immune checkpoint inhibitors is needed.

## Introduction

Current standard of care immunotherapies for melanoma in the adjuvant and metastatic settings include immune checkpoint inhibitors directed at programmed death 1 (PD-1), namely nivolumab and pembrolizumab, and at cytotoxic T-lymphocyte antigen 4 (CTLA-4), ipilimumab, given as monotherapy or in combination. Recently, the Food and Drug Administration approved a third immune checkpoint inhibitor, relatlimab (anti-LAG-3 antibody) in combination with nivolumab for the treatment of patients with metastatic melanoma in the first and later line settings. With the advent of immunological checkpoint inhibitors, the treatment landscape for melanoma has been revolutionized. However, research is ongoing to develop new therapies to further improve outcomes while limiting toxicities.

A recent press release for a phase IIb clinical trial with an mRNA vaccine for melanoma in combination with pembrolizumab in 157 patients with resected stage III or IV melanoma identified a 44% reduction in the risk of recurrence or death when compared to standard of care therapy with pembrolizumab alone (1). This is an exciting development in which the mRNA cancer vaccine can prime patients’ immune system to generate a response to melanoma. However, developing vaccines for melanoma has been ongoing for decades, and the potential for whole cell vaccination strategies requiring further study. Initial vaccine studies included allogeneic and autologous granulocyte-macrophage colony stimulating factor (GM-CSF)-secreting tumor vaccines for pancreas cancer and melanoma (2, 3). Phase I studies demonstrated that GM-CSF secreting vaccines induced delayed-type hypersensitivity responses to autologous tumor cells and that vaccination sites showed brisk infiltrates of dendritic cells, macrophages, eosinophils, and lymphocytes contributing to enhanced tumor antigen presentation and ultimately promoting anti-tumor immunity (3–9). We previously conducted a phase I study with irradiated, autologous melanoma cells engineered to secrete GM-CSF by adenoviral mediated gene transfer, which demonstrated safety for patients and augmentation in anti-tumor immunity (6). Here, we present data from a phase II trial of irradiated, autologous melanoma cells engineered to secrete GM-CSF by adenoviral mediated gene transfer in patients with stage III and IV melanoma.

## Methods

### Patients

Patients were eligible if they were 18 years of age or older and had previously treated or untreated, histologically confirmed, stage III or IV melanoma with ECOG performance status 0 or 1. Patients with stage III melanoma were eligible if gross lymphadenopathy of at least 2cm was present by physical exam or on CT in a region draining a known primary melanoma; and have refused, failed or not been appropriate candidates for adjuvant high-dose interferon. Patients who received prior systemic chemotherapy, radiotherapy, immunotherapy or glucocorticoid therapy were eligible if the last dose was received at least 4 weeks prior to trial enrollment. Patients with prior bone marrow or peripheral blood stem cell transplant were eligible if they were greater than 6 months from transplant at time of trial enrollment. Key exclusion criteria were uveal melanoma, uncontrolled active infection, pregnant or nursing mothers, and infection with HIV. Full inclusion and exclusion criteria are listed in the study protocol. The study protocol was reviewed and approved by the Dana-Farber/Harvard Cancer Center institutional review board. All patients provided written informed consent. An independent data monitoring committee provided oversight to assess efficacy and safety of lethally irradiated, autologous melanoma cells engineered by adenoviral mediated gene transfer to secrete GM-CSF (NCT #00809588).

### Trial design, vaccine preparation and administration

In this phase 2 trial, patients received individual vaccine doses of lethally irradiated, autologous melanoma cells engineered by adenoviral mediated gene transfer to secrete GM-CSF. Methods of vaccine production have been previously described (5). Briefly, excised melanoma metastases were processed to single cells, transduced with a replication defective adenoviral vector encoding human GM-CSF, irradiated with 10,000 cGy, and cryopreserved in liquid nitrogen. GM-CSF secretion was determined by ELISA. A portion of tumor cells was not transduced and used in delayed-type hypersensitivity analysis. Individual vaccines were composed of 1x10^6^, 4x10^6^, 1x10^7^ tumor cells, or one-sixth of total depending upon overall yield, and were injected intradermally (0.5ml) and subcutaneously (0.5ml) into limbs or abdomen on a rotating basis on days 0, 7, 14 and every two weeks thereafter until the supply of vaccine was exhausted or the patient was removed from study. Administration of non-transduced, irradiated cells (1x10^6^) were given on day 0 and with the fifth vaccination intradermally (0.5ml) for evaluation of baseline and vaccine induced delayed-type hypersensitivity. Patients could participate in a second round of tumor procurement, vaccine production, and vaccination as long as the patient continued to meet eligibility criteria. Treatment continued until the occurrence of disease progression, unacceptable adverse effects, or withdrawal of consent. Patients underwent scans at week 10 and then at four-month intervals and peripheral blood was collected for immunologic analysis monthly. Biopsies were performed for vaccination reaction (after first vaccine dose) and delayed-type hypersensitivity reaction 2-3 days after administration and again after fifth vaccine dose for vaccination reaction, and if available, for a second delayed-type hypersensitivity reaction. Please see **Supplementary Figure 1** for clinical trial schema.

### End points and assessments

This study consisted of two parallel cohorts: patients with stage III melanoma and patients with stage IV melanoma. The primary endpoint in the first cohort was feasibility of preparing lethally irradiated, autologous melanoma cells engineered by adenoviral mediated gene transfer to secrete GM-CSF in patients with stage III melanoma. The primary endpoint in the second cohort was to determine the proportion of patients alive at two years. Secondary endpoints for both cohorts included progression free survival, overall survival, and rate of adverse events. Progression-free survival was assessed according to RECIST, version 1.1, by blinded independent review. Adverse events were assessed continuously throughout the trial and for at least 30 days after treatment was discontinued and were graded according to the National Cancer Institute Common Terminology Criteria for Adverse Events, version 2.0. Exploratory endpoints included analysis of cytokines by Luminex platform to analyze correlation of certain biomarkers with clinical outcomes.

The distributions of OS and PFS are presented using the method of Kaplan-Meier with 95% confidence intervals estimated using log[-log(endpoint)] methodology and log-rank testing. All statistical testing is two-sided with nominal significance levels of 0.05. There are no corrections for multiple comparisons. Analyses were performed using SAS 9.4 (SAS Institute Inc., Cary, NC, USA).

### Immunologic analysis

Cytokines and chemokines were quantified in serum samples using the FLEXMAP3D Luminex platform, and the xPONENT software for standard curve extrapolation. The following analytes were analyzed: MMP-1, TRAIL, TSLP, MIF, LIF, MDC, APRIL, TWEAK, CCL-2, CCL-3, CCL-4, CCL-8, CCL-11, CCL-24, CXCL-5, CXCL-10, CXCL-11, CXCL-13, CD30, CD40L, CTLA-8, IL-2, IL-15, IL-16, IL-18, IL-20, SCF, G-CSF, HGF, SDF-1a, TNF-RII, and VEGF-A. Concentrations of analytes below LLOQ were not considered in the analysis. Serum samples were diluted by two and processed for analyses as recommended by manufacturer protocol (Bio-Techne, Minneapolis MN) (10–12).

The endpoint of interest for the Luminex analysis in the Stage III cohort was PFS; for the Stage IV cohort the endpoints were clinical benefit rate (best response of CR, PR, or SD per RECIST 1.1) and PFS. Pretreatment measurements are summarized descriptively; each pretreatment biomarker is divided at its respective median and subsequent PFS summarized stratified by pretreatment high/low using the method of Kaplan-Meier. Changes at four to eight weeks relative to pretreatment are expressed as fold-changes (post/pre) and are summarized descriptively and compared with one using Wilcoxon signed-rank tests. Fold-changes are also divided into high/low according to the respective median of the fold-change. The distributions of subsequent PFS are described using the method of Kaplan-Meier and compared using log-rank tests. Comparisons of clinical benefit rates according to high/low pre-treatment biomarker levels used Fisher’s exact tests. The STROBE cohort reporting guidelines were used (13).

## Results

### Patients

From November 2003 through June 2009, a total of 84 patients were enrolled in this trial to receive a vaccine with lethally irradiated, autologous melanoma cells engineered to secrete GM-CSF. Four patients cancelled registration and nineteen patients did not receive any treatment; the final cohort was comprised of 61 patients, of which 20 had stage III disease and 41 had stage IV disease. The median follow up was 74 months (95%CI, 53 to 116). Overall, the median age was 56.6 years (range 14.4-80.2) with equal distribution of female (28 patients, 45.9%) to male, and predominantly Caucasian (100%) (**Table 1**). Across both stages, a total of 22 patients (36.1%) received prior adjuvant interferon, 12 patients (19.7%) received prior chemotherapy, and 16 patients (26.2%) received prior immunotherapy. The most frequent site of metastases was lymph nodes (36 patients, 59.0%), followed by lung (29 patients, 47.5%) and skin (18 patients, 29.5%) or other sites (19 patients, 31.1%).

**Table 1:** Patient demographics and disease characteristics at baseline.

Characteristics	All (N=61)	Stage	
		III (N=20)	IV (N=41)
Median (min-max)			
**Age (years)**	56.6 (14.4-80.2)	58.5 (26.8-80.2)	54.6 (14.4-77.6)
**BMI**	27.0 (18.7-46.9)	27.9 (19.2-46.9)	26.7 (18.7-34.5)
N (%)			
Gender			
Female	28 (45.9%)	6 (30.0%)	22 (53.7%)
Race			
Caucasian	61 (100.0%)	20 (100.0%)	41 (100.0%)
Ethnicity			
Hispanic or Latino	5 (8.2%)	1 (5.0%)	4 (9.8%)
Non-Hispanic	56 (91.8%)	19 (95.0%)	37 (90.2%)
ECOG performance status			
0	50 (82.0%)	17 (85.0%)	33 (80.5%)
1	9 (14.8%)	2 (10.0%)	7 (17.1%)
Not applicable	2 (3.3%)	1 (5.0%)	1 (2.4%)
Prior chemotherapy			
No	49 (80.3%)	18 (90.0%)	31 (75.6%)
Yes	12 (19.7%)	2 (10.0%)	10 (24.4%)
Prior adjuvant interferon			
No	39 (63.9%)	13 (65.0%)	26 (63.4%)
Yes	22 (36.1%)	7 (35.0%)	15 (36.6%)
Prior other immunotherapy			
No	45 (73.8%)	18 (90.0%)	27 (65.9%)
Yes	16 (26.2%)	2 (10.0%)	14 (34.1%)
Prior radiation therapy			
No	37 (60.7%)	14 (70.0%)	23 (56.1%)
Yes	23 (37.7%)	5 (25.0%)	18 (43.9%)
Unknown	1 (1.6%)	1 (5.0%)	0 (0.0%)
Location of metastases*			
Skin	18 (29.5%)	5 (25.0%)	13 (31.7%)
Liver	9 (14.8%)	0 (0.0%)	9 (22.0%)
Brain	4 (6.6%)	0 (0.0%)	4 (9.8%)
Lung	29 (47.5%)	0 (0.0%)	29 (70.7%)
Lymph nodes	36 (59.0%)	16 (80.0%)	20 (48.8%)
Other	19 (31.1%)	1 (5.0%)	18 (43.9%)
Site of procurement			
Lung	13 (21.3%)	1 (5.0%)	12 (29.3%)
Lymph nodes	21 (34.4%)	14 (70.0%)	7 (17.1%)
Skin	10 (16.4%)	4 (20.0%)	6 (14.6%)
Other	17 (27.9%)	1 (5.0%)	16 (39.0%)

*Some patients may have more than one site of metastatic disease.

### Efficacy

Among all patients, the median number of vaccinations was 7 (range 1-27). Among patients with stage IV disease, 1 patient (2.4%) had a partial response, 11 patients had stable disease (26.8%), and 29 patients (70.7%) had progressive disease (**Table 2**). Of patients with stage III disease, 14 (70%) had no evidence of disease and 3 (15%) had progressive disease. In the total cohort, the disease control rate (CR+PR+SD) was 39.3% (95%CI, 27.1 to 52.7%).

**Table 2:** Response rates stratified by stage.

Characteristics	All (N=61)	Stage	
		III (N=20)	IV (N=41)
Median (min-max)			
**Total # vaccinations**	7.0 (1.0-27.0)	8.5 (3.0-27.0)	6.0 (1.0-27.0)
N (%)			
Best objective response			
Partial response	1 (1.6%)	0 (0.0%)	1 (2.4%)
Stable disease	23 (37.7%)	0 (0.0%)	11 (26.8%)
Progressive disease	32 (52.5%)	3 (15.0%)	29 (70.7%)
No evidence of disease	2 (3.3%)	14 (70.0%)	0 (0.0%)
Missing	3 (4.9%)	3 (15.0%)	0 (0.0%)
Progression on treatment			
No	27 (44.3%)	16 (80.0%)	11 (26.8%)
Yes	34 (55.7%)	4 (20.0%)	30 (73.2%)
Cause of death			
Progressive disease	37 (60.7%)	9 (45.0%)	28 (68.3%)
Unknown	2 (3.3%)	0 (0.0%)	2 (4.9%)
Alive/Loss to follow-up	22 (36.1%)	11 (55.0%)	11 (26.8%)
Survival status			
Alive	20 (32.8%)	11 (55.0%)	9 (22.0%)
Dead	39 (63.9%)	9 (45.0%)	30 (73.2%)
Loss to follow-up	2 (3.3%)	0 (0.0%)	2 (4.9%)
Reason off-study			
Patient completed protocol treatment	32 (52.5%)	17 (85.0%)	15 (36.6%)
Progressive disease	27 (44.3%)	3 (15.0%)	24 (58.5%)
Other	2 (3.3%)	0 (0.0%)	2 (4.9%)

**Figure 1:**
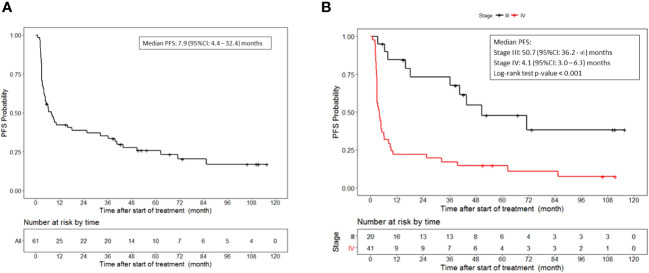
Progression free survival. **(A)** PFS for all patients who received vaccine therapy. **(B)** PFS stratified by stage III or IV melanoma who received vaccine therapy. Tick marks indicate censored data.

With a median follow up of 74 months, the median progression-free survival was 7.9 (95%CI, 4.4 to 32.4) months in patients who received lethally irradiated, autologous melanoma cells engineered by adenoviral mediated gene transfer to secrete GM-CSF (**Figure 1A**). The median progression-free survival for patients with stage III disease was 50.7 (95%CI, 36.2 to NR) months and 4.1 (95%CI, 3.0 to 6.3) months for patients with stage IV disease (p<0.001) (**Figure 1B**).

**Figure 2:**
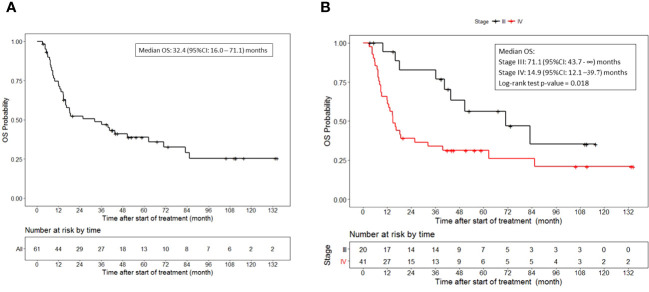
Overall survival. **(A)** OS for all patients who received vaccine therapy. **(B)** OS stratified by stage III or IV melanoma who received vaccine therapy. Tick marks indicate censored data.

The median overall survival was 32.4 (95%CI, 16.0 to 71.1) months (**Figure 2A**). Additionally, the median overall survival for patients with stage III disease was 71.1 (95%CI, 43.7 to NR) months and 14.9 (95%CI, 12.1 to 39.7) months for patients with stage IV disease (p = 0.018) (**Figure 2B**).

### Safety

The most frequent treatment-related adverse events are shown in **Table 3**. Of 61 patients, a total of 58 patients (95.1%) had any treatment-related adverse event, mostly grades 1-2. The most common grade 1 or 2 treatment-related adverse events included erythema in 46 patients (75.4%), injection site reactions in 30 patients (49.2%), and pruritis in 18 patients (29.5%). One patient had a grade 3 treatment-related adverse event of wound infection. No autoimmune reactions or adenoviral infections were observed. All other grade 3 or 4 adverse events were not related to treatment. For a full list of treatment-related adverse events and any adverse event, please see **Supplementary Tables 1** and **2**. Three grade 4 adverse events occurred, unlikely related to treatment. These included abdominal pain, pericarditis, and constitutional.

**Table 3:** Summary of adverse events.

Adverse Event	Any Grade	Grade 1	Grade 2	Grade 3	Grade 4
Any Adverse Event	60 (98.4%)	18 (29.5%)	32 (52.5%)	7 (11.5%)	3 (4.9%)
Treatment-Related	58 (95.1%)	37 (60.7%)	20 (32.8%)	1 (1.6%)	–
Treatment-related with at least 10% incidence					
Erythema	46 (75.4%)	40 (65.6%)	6 (9.8%)	–	–
Injection Site Reaction	30 (49.2%)	25 (41.0%)	5 (8.2%)	–	–
Pruritis	18 (29.5%)	17 (27.9%)	1 (1.6%)	–	–
Pain*	12 (19.7%)	7 (11.5%)	5 (8.2%)	–	–
Fatigue	12 (19.7%)	10 (16.4%)	2 (3.3%)	–	–
Lab Abnormalities†	8 (13.1%)	7 (11.5%)	1 (1.6%)	–	–
Arthralgia	8 (13.1%)	7 (11.5%)	1 (1.6%)	–	–
Myalgia	8 (13.1%)	7 (11.5%)	1 (1.6%)	–	–
Grade 3 or higher, any attribution					
Lab abnormalities†	6 (9.8%)	–	–	6 (9.8%)	–
Edema	1 (1.6%)	–	–	1 (1.6%)	–
Pericarditis	1 (1.6%)	–	–	–	1 (1.6%)
Fatigue	1 (1.6%)	–	–	1 (1.6%)	–
Constitutional	1 (1.6%)	–	–	–	1 (1.6%)
Wound Infection**±**	1 (1.6%)	–	–	1 (1.6%)	–
Metabolic	2 (3.3%)	–	–	2 (3.3%)	–
Pain*	4 (6.6%)	–	–	3 (1.6%)	1 (1.6%)

*Tumor, bone, general, abdominal or joint pain.

†Changes in hemoglobin, leukocyte, platelet counts, LFTs, or electrolytes.

**±** Deemed related to treatment; all other Grade-3+ not related.

### Delayed-type hypersensitivity reactions

Irradiated, autologous nontransduced melanoma cells were available for delayed-type hypersensitivity testing in 60 patients (insufficient cells precluded these studies in one patient). Sixty patients received at least one injection; 88% of patients received at least two injections, for a total of 124 injections in the entire cohort. Most patients received injection at a dose of 1x10^6^ (range: 1x10^6^ to 4x10^6^). About one third of injections provoked a clinical reaction at the injection site. The most common injection site reactions included erythema (33% of injections), induration (35%), redness (20%) and pruritis (9%). **Supplementary Table 5** details delayed-type hypersensitivity (DTH) reactions. No association between a positive DTH reaction and PFS was observed.

### Luminex

#### Stage III patients

**Figure 3:**
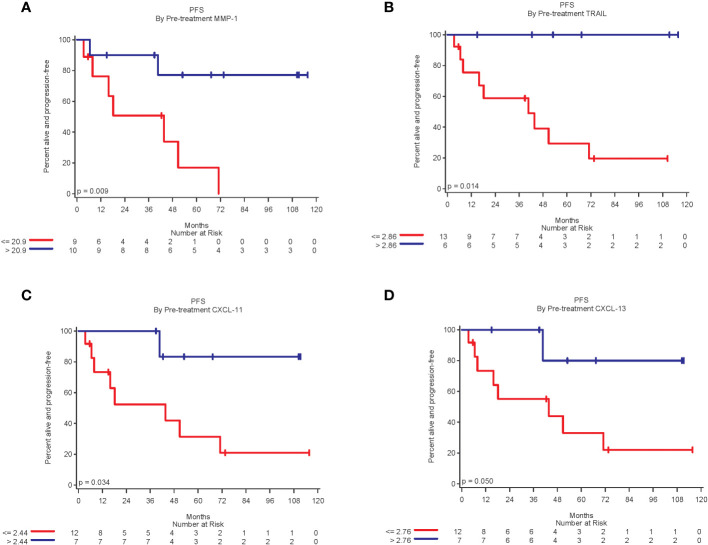
Kaplan-Meier plots of biomarkers related to improved PFS. **(A)** Patients with pre-treatment MMP-1 expression above the median of 20.9 was associated with significantly improved PFS (log-rang p=0.009); **(B)** Patients with TRAIL expression above the median of 2.86 was associated with significantly better PFS (p = 0.01). **(C)** Patients with CXCL-11 expression above median of 2.44 was associated with significantly better PFS (p=0.03); and **(D)** Patients with CXCL-13 expression above the median of 2.76 was associated with significantly better PFS (p = 0.05).

A total of 32 biomarkers were analyzed by Luminex. For stage III patients, 19 had pre-treatment biomarker data and 18 also had biomarker data 4-8 weeks after the first dose of vaccine. In stage III patients, higher pre-treatment levels of MMP-1, TRAIL, CXCL-11, and CXCL-13 were statistically significantly related to improved PFS (p<0.05 for all), (**Figure 3**). Higher pre-treatment levels of CD40L, CTLA-8, and IL-20 trended toward improved PFS, but were not statistically significant (p=0.09, p=0.07 and p=0.08, respectively).

IL-16 and IL-20 had statistically significant increases in expression during the first 4-8 weeks after first vaccine when compared to pre-treatment levels (median increase of 76% and 58%, respectively; p<0.05 for both). For a complete list of biomarkers comparing fold-change from pre-treatment to 4-8 weeks post first vaccine, please see **Supplementary Table 3**.

**Figure 4:**
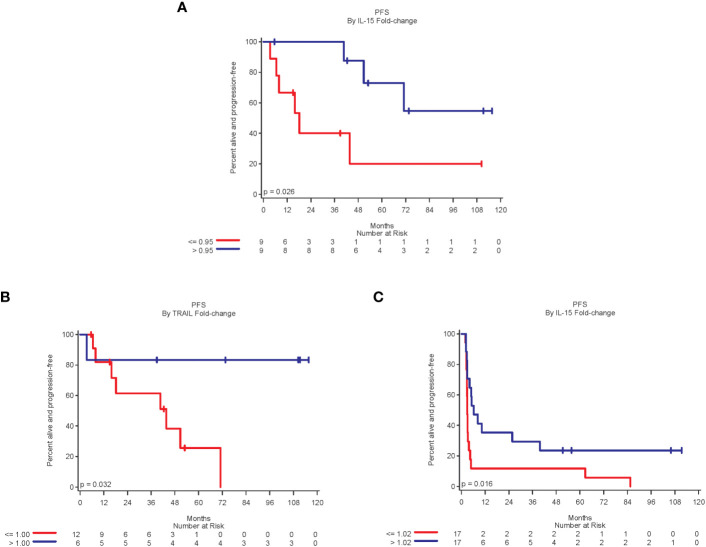
A fold-change above the median of 0.95 for IL-15 [**(A)**, Stage III]; 1.00 for TRAIL [**(B)**, Stage III]; and 1.02 for IL-16 [**(C)**, Stage IV] was significantly associated with improved PFS.

In an exploratory analysis of 18 stage III patients, the fold-change of IL-15 and TRAIL levels when comparing pre-treatment to 8 weeks after first vaccine were significantly associated with PFS. A 5% or more decrease in IL-15 was associated with worse PFS compared to patients who had an increase in IL-15 (p=0.03) (**Figure 4A**). Similarly, an increase in fold-change of TRAIL was significantly associated with improved PFS (p=0.03) (**Figure 4B**). A decrease in fold-change in CXCL-11 demonstrated improved PFS but did not reach statistical significance (p=0.054).

#### Stage IV patients

For stage IV disease, 37 patients had pre-treatment biomarker data and 36 patients had biomarker data 4-8 weeks after the first vaccine dose. No statistically significant associations were demonstrated between pre-treatment biomarker levels and PFS. Lower pre-treatment levels of MDC and VEGF-A demonstrated better PFS, although not statistically significant (p=0.12 and p=0.14, respectively).

In comparing fold-change of cytokine expression from pre-treatment levels to 4-8 weeks after first vaccine, CTLA-8 and CXCL-5 demonstrated a statistically significant decrease (p=0.02 and p=0.01, respectively) (**Supplementary Table 4**). MMP-1 and IL-18 demonstrated decrease in fold-change, although not statistically significant (p=0.09 and p=0.07, respectively).

Increase in fold-change of IL-16 after treatment was significantly associated with improved PFS when compared to pre-treatment levels (p=0.02) (**Figure 4C**). Increases in CCL-4 and CXCL-11 during the first 4-8 weeks were associated with improved PFS, although not statistically significant (p=0.08 for both). A greater than 30% decrease in CCL-24 levels was associated with worse PFS, although not statistically significant (p=0.07). A fold-change in IL-16 was also related to clinical benefit. The clinical benefit rate (CR, PR, or SD) in patients with more than 2% increase in IL-16 was 47% (8 of 17 patients), compared to 12% in patients with a decrease or small increase in IL-16.

For stage IV patients, 11 patients experienced a DTH reaction and had pre-treatment Luminex data available and 23 did not have a DTH reaction but had Luminex data available. Pretreatment CXCL-11 levels were higher in patients who experienced a DTH reaction compared to patients who did not have a DTH reaction, although not statistically significant (p=0.10). CXCL-5 levels were lower in patients who had a DTH reaction compared to those that did not have a DTH reaction but was not statistically significant (p = 0.08). At a median of four weeks after first vaccine, 32 patients with stage IV disease had available Luminex data for analysis, of which 10 patients had a DTH reaction. CTLA-8 levels were lower in patients who had a reaction, although not statistically significant (p=0.08). **Supplementary Table 6** details Luminex data for patients with and without DTH reaction.

## Discussion

This phase II trial of autologous GM-CSF-secreting melanoma cell vaccines in stage III and IV patients demonstrates modest efficacy with evidence for the enhancement of anti-tumor immunity. The disease control rate in the overall cohort was 39.3% in patients who received GM-CSF secreting vaccines. Four of twenty (20%) patients with stage III disease progressed while receiving treatment, with a median PFS of 4.2 years and median OS of 5.9 years (5-year OS rate of 62%). Historically, the median recurrence free survival was about 2.2 to 2.5 years for resected high-risk stage III-IV patients receiving adjuvant interferon and median 5-year OS rate was 60% (14–17). This is comparable or inferior to our findings with adjuvant GM-CSF vaccine, which pre-dates the era of immune checkpoint inhibitor therapy. Vaccination is also associated with less toxicities compared to interferon-alpha (14–17).

Thirty of forty-one (72.2%) patients with stage IV disease progressed while receiving treatment, with a median PFS of 4.1 months and median OS of 14.9 months. These findings are comparable to historic data for ipilimumab in patients with metastatic disease, with median OS of 11.4 months in patients receiving ipilimumab for metastatic melanoma (18). Together, our data suggest that the GM-CSF secreting vaccine may be more efficacious in patients with locoregional disease compared to metastatic disease. Prior studies have demonstrated that functional tumor-specific T cells are more frequently found in melanoma patients with regional metastasis (71%), in comparison to patients with distant metastasis (23%) (19). Additionally, the greater efficacy of GM-CSF vaccination in the adjuvant setting may result in eradication of residual micro-metastasis if present, and higher frequencies of functional tumor-specific T cells may be due to lower tumor burden and ultimately less tumor-mediated immune suppression compared to metastatic disease (20).

In addition, the results of Luminex analysis demonstrated stronger correlation to changes in anti-tumor immunity for stage III patients. Higher pre-treatment levels of MMP-1, TRAIL, CXCL-11, and CXCL-13 were associated with improved PFS. Matrix metalloproteinase-1 (MMP-1) serves as an extracellular matrix degrading enzyme that facilitates tumor migration and invasion, promoting melanoma growth and metastasis (21). Higher levels of MMP-1 prior to vaccination might, however, modify immune cell trafficking or modulate dendritic cell function to facilitate an anti-tumor immune response. Similarly, TNFα-related apoptosis-inducing ligand (TRAIL) may promote extrinsic proapoptotic pathways through death receptor mediated signaling, potentially stimulating tumor cell killing in the presence of immune activation (22–25). Additionally, the presence of inflammatory (CXCL-11) and lymphoid (CXCL-13) chemokines has been associated with the recruitment of tumor infiltrating lymphocytes, namely CD4+ T cells, CD8+ T cells and mature dendritic cells, to promote melanoma cell destruction (26–28). CXCL-13 is also linked to the formation of tertiary lymphoid structures, and prior work showed that GM-CSF based vaccines stimulate coordinated T cell and antibody responses (4, 6). Together, these data raise the possibility that vaccine efficacy might be influenced, at least in part, by particular mixtures of chemokines and soluble tumor-associated factors that are present at the time of initiating therapy.

Luminex results for stage III patients also demonstrated increased expression of IL-16 and IL-20 after vaccine therapy, which suggests the initiation and/or amplification of an inflammatory response via activation of CD4+ T cells that might secrete additional pro-inflammatory cytokines including IL-1β, IL-6 and TNF-α (29–31). Moreover, increases in expression of IL-15 and TRAIL after vaccine therapy were associated with improved PFS in stage III patients. Recent studies have suggested that interleukins, like IL-15 elicit changes in natural killer cells that subsequently provide anti-tumor functionality and can remain active for weeks after first cytokine stimulation (32, 33). This further suggests that inflammatory cytokines induced by vaccination potentially contribute to tumor cell destruction and anti-tumor immunity.

Stage IV patients similarly demonstrated increases in IL-16 after vaccination that were associated with improved PFS and clinical benefit. IL-16 stimulates the production of pro-inflammatory cytokines, which would also support anti-tumor immunity (30). Levels of CTLA-8 (IL-17) and CXCL-5 decreased after vaccination, potentially indicating a reduction in tumor-promoting inflammation. Indeed, several studies have indicated that CTLA-8 (IL-17) may stimulate cancer cells to produce angiogenic factors like VEGF, thereby enhancing tumor angiogenesis and growth via STAT3 signaling (34–37). While CXCL-5 is a chemokine that recruits and activates leukocytes, it also promotes angiogenesis, tumor growth, and metastasis (38–41). High levels of CXCL-5 have similarly been associated with clinical response in a small study of patients treated with ipilimumab plus nivolumab, suggesting that the role of this chemokine in immunotherapy should be investigated in more detail (42).

The use of GM-CSF in cancer vaccines is based on the ability of the cytokine to increase antigen-specific immune responses and to function as an immune adjuvant for dendritic cells (43). Indeed, autologous dendritic cell vaccines can generate potent anti-tumor immune responses via enhanced tumor antigen presentation (44). The median OS demonstrated in our study of 14.9 months for stage IV patients is similar to prior dendritic cell vaccine trials using allogeneic and autologous tumor cells, with OS ranging from 12 to 14 months (45, 46). Moreover, Dillman et al. conducted a randomized phase II trial that compared autologous tumor cell vaccine (TC) with autologous tumor cells loaded onto dendritic cells (DCV) admixed with GM-CSF protein in 42 patients with metastatic melanoma (47–49). This trial demonstrated that the DCV arm achieved a superior 2-year survival rate of 72% versus 31% in the TC arm (p=0.007) (48). At 5-years, patients who received DCV survived longer with a median OS of 43.4 months versus 20.5 months, and showed a 70% reduction in the risk of death (HR 0.30, p=0.005) (49). Similarly, two clinical trials that administered ipilimumab to patients with metastatic melanoma or ovarian carcinoma that were previously vaccinated with autologous GM-CSF-secreting melanoma or ovarian cancer vaccines demonstrated extensive tumor necrosis or the reduction of cancer antigen-125 (CA-125) levels (50, 51). These findings suggest that the addition of CTLA-4 inhibition might intensify tumor immunity in patients who have been previously vaccinated. In this context, GM-CSF secreting melanoma vaccines may serve to prime a patient’s immune system, whereas the addition of checkpoint blockade or other immune activating mechanisms may augment the anti-tumor response and potentiate clinical efficacy.

Additional promising investigations in vaccine therapy include neoantigen vaccines in melanoma. Several studies with neoantigen vaccines using mRNA or peptides admixed with poly-ICLC (NeoVax) were recently published (52–54). Ott et al. demonstrated in six high-risk resected stage III-IV melanoma patients that personalized vaccines formulated with up to 20 predicted MHC class I restricted neoantigens induced specific CD4+ and, to a lesser extent, CD8+ T cells (54). At 25 months after vaccination, four patients showed no recurrence and the two patients who did recur subsequently achieved complete responses after anti-PD-1 therapy. mRNA vaccines similarly demonstrated the generation of antigen specific CD4+ and CD8+ T cells and a phenomenon of epitope-spreading (52–54). Neoantigen vaccines are now being broadly studied in conjunction with anti-PD-1 therapies in several disease settings.

The current study demonstrates the feasibility of an autologous whole cell vaccination strategy in stage III and stage IV metastatic melanoma patients. Limitations of this study include the small cohort size and the single-arm design. Since there was no comparator cohort, we cannot definitively demonstrate an improvement in PFS or OS that can be attributed to GM-CSF secreting melanoma vaccines. Follow up data on subsequent therapies that patients received after vaccination were not collected, so the potential impact of immune checkpoint inhibitor therapy on PFS or OS is unknown at this time. The Luminex findings are also exploratory and must be verified in a larger cohort of patients. Additionally, biopsies of metastatic sites may have provided further information on immune cell infiltration, especially in comparison to local vaccine site biopsies. Prior studies have demonstrated infiltrates composed of dendritic cells, macrophages, T and B lymphocytes, and eosinophils in vaccination sites and distant metastases (4, 6). Lastly, the efficacy of such a vaccination strategy may be of greater benefit in patients with limited disease in the adjuvant setting and with the use of immune checkpoint blockade.

In conclusion, this study demonstrates that vaccination with irradiated, autologous melanoma cells engineered to secrete GM-CSF may elicit antitumor immunity in patients with stage III and IV melanoma. The overall disease control rate of 39%, with a median follow up of 74 months, is intriguing and raises the possibility that GM-CSF vaccination might be effectively combined with immune checkpoint inhibitor therapy. The median PFS of 50.7 months and median OS of 71.1 months in high-risk resected stage III patients is comparable to or potentially superior in efficacy to interferon in the pre-ICI era and suggests that GM-CSF vaccination may be particularly efficacious when combined with immune checkpoint blockade for stage III patients. The prolonged overall survival in stage III patients suggests that the presence of minimal/locoregional disease might facilitate the priming of anti-tumor immunity with GM-CSF secreting melanoma vaccines, which might be further intensified with the addition of immune checkpoint inhibitors.

## Data availability statement

The original contributions presented in the study are included in the article/**Supplementary Material**. Further inquiries can be directed to the corresponding author.

## Ethics statement

The studies involving humans were approved by Dana-Farber/Harvard Cancer Center Institutional Review Board. The studies were conducted in accordance with the local legislation and institutional requirements. The participants provided their written informed consent to participate in this study.

## Author contributions

TS: Writing – original draft, Writing – review & editing. MS: Data curation, Investigation, Methodology, Writing – review & editing. AG-H: Formal analysis, Methodology, Validation, Writing – review & editing. PF: Investigation, Validation, Writing – review & editing. SS: Investigation, Writing – review & editing. MJ: Investigation, Writing – review & editing. TC: Investigation, Writing – review & editing. LG: Investigation, Writing – review & editing. DL: Investigation, Writing – review & editing. RS: Investigation, Writing – review & editing. HD: Project administration, Writing – review & editing. JR: Investigation, Supervision, Validation, Writing – review & editing. GD: Investigation, Methodology, Supervision, Visualization, Writing – review & editing. FH: Investigation, Methodology, Resources, Supervision, Validation, Visualization, Writing – review & editing.
