# Supplementary figures and images for: Phase II trial of vaccination with autologous, irradiated melanoma cells engineered by adenoviral mediated gene transfer to secrete granulocyte-macrophage colony stimulating factor in patients with stage III and IV melanoma
Source: Front Oncol. 2024 May 15;14:1395978. doi: 10.3389/fonc.2024.1395978 (PMC11133610; doi:10.3389/fonc.2024.1395978)

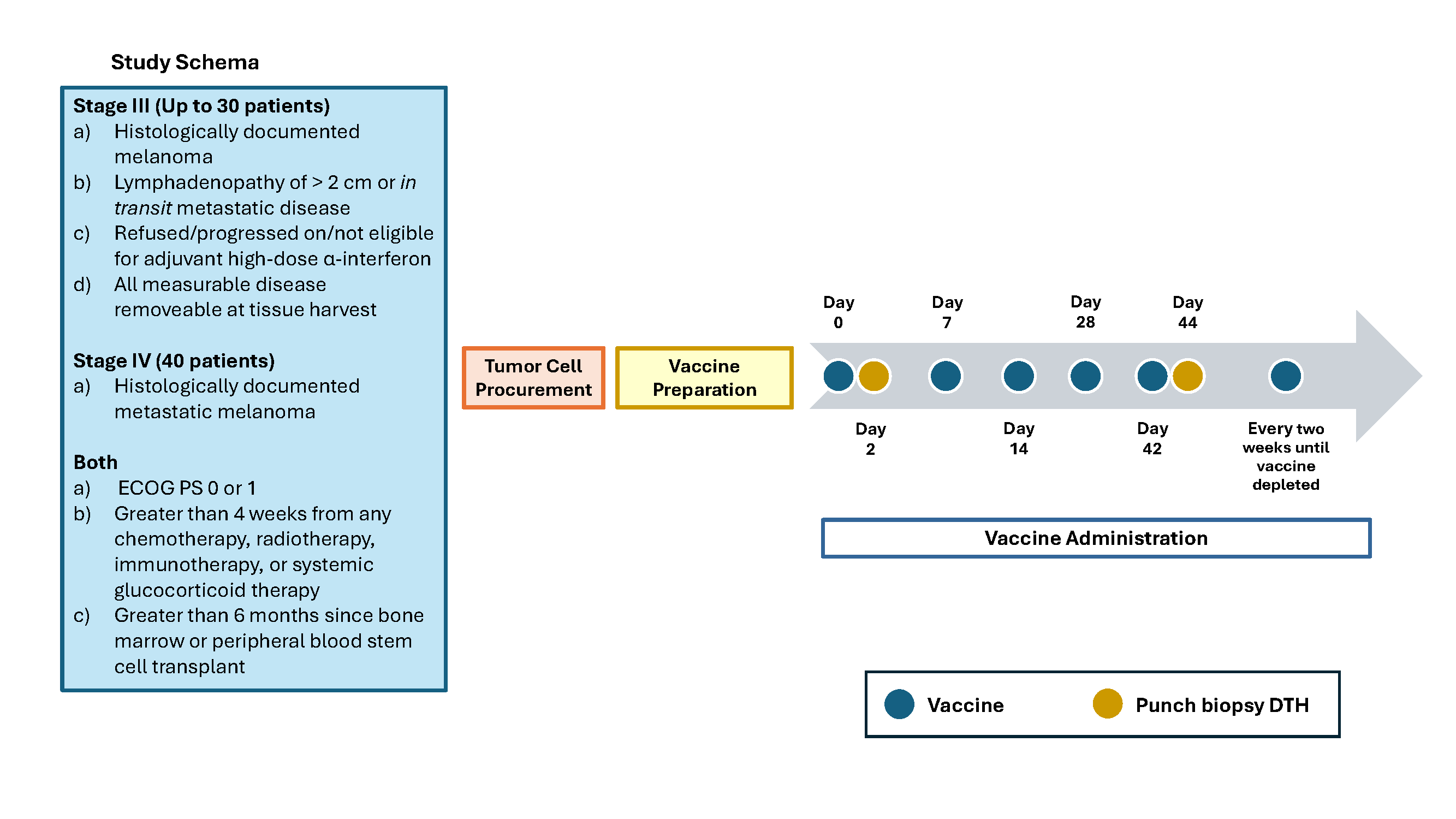

Supplement: Supplementary file 1 [file Image_1.tiff]
