# Supplementary material for: Phase II trial of vaccination with autologous, irradiated melanoma cells engineered by adenoviral mediated gene transfer to secrete granulocyte-macrophage colony stimulating factor in patients with stage III and IV melanoma
Source: Front Oncol. 2024 May 15;14:1395978. doi: 10.3389/fonc.2024.1395978 (PMC11133610; doi:10.3389/fonc.2024.1395978)
Supplement: Supplementary file 2 [file Table_1.docx]

**Supplemental Table 1. Treatment-related toxicity events by maximum grade (N=58)**

|  | **Grade** | | |
| --- | --- | --- | --- |
| **Description of toxicity** | **1** | **2** | **3** |
| Erythema | 40 | 6 | 0 |
| Injection site reaction | 25 | 5 | 0 |
| Pruritus | 17 | 1 | 0 |
| Fatigue | 10 | 2 | 0 |
| Arthralgia | 7 | 1 | 0 |
| Myalgia | 7 | 1 | 0 |
| Pain-other | 4 | 2 | 0 |
| Leukocytes | 4 | 0 | 0 |
| Hematologic-other | 4 | 0 | 0 |
| Hemoglobin | 3 | 0 | 0 |
| Bone pain | 2 | 1 | 0 |
| Nausea | 2 | 0 | 0 |
| Alkaline phosphatase | 2 | 0 | 0 |
| SGPT | 2 | 0 | 0 |
| Tumor pain | 1 | 1 | 0 |
| Skin-other | 1 | 1 | 0 |
| Anorexia | 1 | 1 | 0 |
| Insomnia | 1 | 1 | 0 |
| Edema | 2 | 0 | 0 |
| Anxiety/agitation | 1 | 1 | 0 |
| Wound - infectious | 0 | 1 | 1 |
| Lymphatics-other | 2 | 0 | 0 |
| Headache | 1 | 1 | 0 |
| Transfusion: pRBCs | 1 | 0 | 0 |
| Hyperkalemia | 1 | 0 | 0 |
| Abdominal pain | 1 | 0 | 0 |
| Hyperglycemia | 1 | 0 | 0 |
| Metabolic-other | 1 | 0 | 0 |
| Joint, muscle, bone-other | 0 | 1 | 0 |
| Lymphopenia | 0 | 1 | 0 |
| Vertigo | 1 | 0 | 0 |
| Fever | 1 | 0 | 0 |
| SGOT | 1 | 0 | 0 |
| Constitutional | 1 | 0 | 0 |
| Rash/desquamation | 1 | 0 | 0 |
| Endocrine-other | 1 | 0 | 0 |
| Constipation | 1 | 0 | 0 |
| Platelets | 1 | 0 | 0 |
| Reproductive function-other | 1 | 0 | 0 |
| Rigors/chills | 1 | 0 | 0 |
| Urticaria | 1 | 0 | 0 |
| Sweating | 1 | 0 | 0 |
| Dizziness/lightheadedness | 1 | 0 | 0 |
| Wound - non-infectious | 1 | 0 | 0 |

**Supplemental Table 2. Toxicity events with all relatedness by maximum grade (N=60)**

|  | **Grade** | | | |
| --- | --- | --- | --- | --- |
| **Description of toxicity** | **1** | **2** | **3** | **4** |
| Erythema | 41 | 6 | 0 | 0 |
| Hyperglycemia | 25 | 6 | 1 | 0 |
| Injection site reaction | 25 | 5 | 0 | 0 |
| Fatigue | 21 | 7 | 1 | 0 |
| Hemoglobin | 19 | 3 | 0 | 0 |
| Pruritus | 18 | 1 | 0 | 0 |
| Metabolic-other | 14 | 3 | 2 | 0 |
| Pain-other | 8 | 7 | 1 | 0 |
| Leukocytes | 13 | 3 | 0 | 0 |
| Arthralgia | 10 | 5 | 0 | 0 |
| SGPT | 13 | 2 | 0 | 0 |
| Myalgia | 11 | 3 | 0 | 0 |
| Alkaline phosphatase | 8 | 3 | 2 | 0 |
| Abdominal pain | 8 | 2 | 1 | 1 |
| Edema | 8 | 3 | 1 | 0 |
| SGOT | 8 | 2 | 1 | 0 |
| Hematologic-other | 11 | 0 | 0 | 0 |
| Cough | 10 | 0 | 0 | 0 |
| Nausea | 6 | 1 | 0 | 0 |
| Hypoglycemia | 7 | 0 | 0 | 0 |
| Anxiety/agitation | 4 | 2 | 0 | 0 |
| Hyperkalemia | 6 | 0 | 0 | 0 |
| Headache | 5 | 1 | 0 | 0 |
| Fever | 5 | 0 | 0 | 0 |
| Constipation | 4 | 1 | 0 | 0 |
| Hepatic-other | 4 | 1 | 0 | 0 |
| Rash/desquamation | 5 | 0 | 0 | 0 |
| Tumor pain | 2 | 2 | 1 | 0 |
| Dizziness/lightheadedness | 5 | 0 | 0 | 0 |
| Anorexia | 2 | 2 | 0 | 0 |
| Vomiting | 3 | 1 | 0 | 0 |
| Insomnia | 3 | 1 | 0 | 0 |
| Creatinine | 4 | 0 | 0 | 0 |
| Dyspnea | 2 | 2 | 0 | 0 |
| Bilirubin | 2 | 2 | 0 | 0 |
| Bicarbonate | 4 | 0 | 0 | 0 |
| Bone pain | 2 | 2 | 0 | 0 |
| Platelets | 3 | 0 | 1 | 0 |
| Hypocalcemia | 4 | 0 | 0 | 0 |
| Weight loss | 3 | 0 | 0 | 0 |
| Constitutional | 2 | 0 | 0 | 1 |
| GI-other | 3 | 0 | 0 | 0 |
| Lymphatics-other | 2 | 1 | 0 | 0 |
| Skin-other | 2 | 1 | 0 | 0 |
| Hypomagnesemia | 2 | 0 | 1 | 0 |
| Urticaria | 3 | 0 | 0 | 0 |
| Hypokalemia | 3 | 0 | 0 | 0 |
| Wound - infectious | 1 | 1 | 1 | 0 |
| Diarrhea w/o prior colostomy | 1 | 1 | 0 | 0 |
| Hyperuricemia | 2 | 0 | 0 | 0 |
| Joint, muscle, bone-other | 0 | 2 | 0 | 0 |
| Dysuria | 2 | 0 | 0 | 0 |
| Lymphopenia | 1 | 1 | 0 | 0 |
| Infection w/o neutropenia | 0 | 2 | 0 | 0 |
| Pulmonary-other | 2 | 0 | 0 | 0 |
| Vertigo | 2 | 0 | 0 | 0 |
| Sweating | 2 | 0 | 0 | 0 |
| Pigmentation | 0 | 2 | 0 | 0 |
| Rigors/chills | 2 | 0 | 0 | 0 |
| Renal/GU-other | 2 | 0 | 0 | 0 |
| Hypermagnesemia | 2 | 0 | 0 | 0 |
| Blurred vision | 2 | 0 | 0 | 0 |
| Allergy-other | 2 | 0 | 0 | 0 |
| Hyponatremia | 2 | 0 | 0 | 0 |
| Hematuria | 1 | 0 | 0 | 0 |
| Depression | 0 | 1 | 0 | 0 |
| Proteinuria | 1 | 0 | 0 | 0 |
| Transfusion: pRBCs | 1 | 0 | 0 | 0 |
| Hypoalbuminemia | 1 | 0 | 0 | 0 |
| Urinary retention | 1 | 0 | 0 | 0 |
| Vasovagal episode | 0 | 1 | 0 | 0 |
| Neuropathy-sensory | 1 | 0 | 0 | 0 |
| Missing | 1 | 0 | 0 | 0 |
| Voice changes/stridor | 1 | 0 | 0 | 0 |
| Vaginal bleeding | 1 | 0 | 0 | 0 |
| Coagulation-other | 1 | 0 | 0 | 0 |
| Hypophosphatemia | 1 | 0 | 0 | 0 |
| Allergic rhinitis | 1 | 0 | 0 | 0 |
| Hypercalcemia | 1 | 0 | 0 | 0 |
| Endocrine-other | 1 | 0 | 0 | 0 |
| Urinary frequency/urgency | 1 | 0 | 0 | 0 |
| Hearing-other | 1 | 0 | 0 | 0 |
| Pain due to radiation | 0 | 1 | 0 | 0 |
| Bruising | 1 | 0 | 0 | 0 |
| Chest pain | 0 | 1 | 0 | 0 |
| Gastritis | 1 | 0 | 0 | 0 |
| Infection-other | 1 | 0 | 0 | 0 |
| Epistaxis | 1 | 0 | 0 | 0 |
| Neuropathic pain | 1 | 0 | 0 | 0 |
| Reproductive function-other | 1 | 0 | 0 | 0 |
| Hemoptysis | 1 | 0 | 0 | 0 |
| Syndromes-other | 1 | 0 | 0 | 0 |
| Rectal bleeding | 1 | 0 | 0 | 0 |
| Hypotension | 1 | 0 | 0 | 0 |
| Pericardial effusion/pericarditis | 0 | 0 | 0 | 1 |
| Flatulence | 1 | 0 | 0 | 0 |
| Arthritis | 0 | 1 | 0 | 0 |
| Neutrophils | 1 | 0 | 0 | 0 |
| CPK | 1 | 0 | 0 | 0 |
| Wound - non-infectious | 1 | 0 | 0 | 0 |
| Hypertension | 1 | 0 | 0 | 0 |
| Hypernatremia | 1 | 0 | 0 | 0 |
