# Supplementary material for: Phase II trial of vaccination with autologous, irradiated melanoma cells engineered by adenoviral mediated gene transfer to secrete granulocyte-macrophage colony stimulating factor in patients with stage III and IV melanoma
Source: Front Oncol. 2024 May 15;14:1395978. doi: 10.3389/fonc.2024.1395978 (PMC11133610; doi:10.3389/fonc.2024.1395978)
Supplement: Supplementary file 3 [file Table_2.docx]

**Supplemental Table 3. Fold change in biomarkers from pre-treatment to 4-8 weeks after initial vaccine dose for stage III patients.**

| Biomarker | N | Mean | Std | Min | Median | Max | p-value |
| --- | --- | --- | --- | --- | --- | --- | --- |
| MCP_2_CCL8 | 18 | 1.02 | 0.55 | 0.05 | 1.01 | 2.14 | 0.99 |
| IL_2R | 18 | 2.88 | 5.98 | 0.00 | 1.00 | 20.34 | 0.89 |
| MIP_1_alpha_CCL3 | 18 | 16.43 | 63.03 | 0.00 | 1.04 | 268.91 | 0.22 |
| SDF_1_alpha | 18 | 129.64 | 536.58 | 0.00 | 1.00 | 2279.6 | 0.38 |
| LIF | 18 | 1.33 | 1.47 | 0.00 | 0.98 | 6.51 | 0.93 |
| IP_10_CXCL10 | 18 | 1.11 | 0.91 | 0.04 | 1.14 | 4.39 | 0.80 |
| BLC_CXCL13 | 18 | 3.76 | 12.74 | 0.00 | 1.00 | 54.78 | 0.38 |
| Eotaxin_2_CCL24 | 18 | 1.05 | 0.77 | 0.00 | 1.00 | 3.08 | 0.98 |
| Eotaxin_CCL11 | 18 | 1.63 | 2.90 | 0.01 | 0.98 | 13.04 | 0.90 |
| IL_17A_CTLA_8 | 18 | 1.41 | 1.94 | 0.00 | 0.91 | 8.79 | 0.93 |
| SCF | 18 | 1.09 | 1.13 | 0.00 | 0.77 | 4.56 | 0.51 |
| G_CSF_CSF_3 | 18 | 2.85 | 4.34 | 0.00 | 1.20 | 16.88 | 0.24 |
| HGF | 18 | 1.27 | 1.45 | 0.00 | 1.02 | 6.60 | 0.99 |
| MIP_1_beta_CCL4 | 18 | 1.97 | 3.19 | 0.00 | 1.00 | 10.62 | 0.77 |
| MCP_1_CCL2 | 18 | 6.09 | 16.70 | 0.00 | 0.98 | 70.82 | 0.55 |
| MIF | 18 | 1.85 | 2.97 | 0.00 | 1.17 | 13.18 | 0.35 |
| I_TAC_CXCL11 | 18 | 17.82 | 70.58 | 0.00 | 1.00 | 300.55 | 0.83 |
| TRAIL | 18 | 3.16 | 7.87 | 0.00 | 1.00 | 34.37 | 0.27 |
| MMP_1 | 18 | 8.05 | 30.67 | 0.00 | 0.86 | 130.89 | 0.52 |
| IL_15 | 18 | 0.87 | 0.58 | 0.00 | 0.95 | 1.85 | 0.52 |
| IL_18 | 18 | 1.08 | 0.61 | 0.06 | 0.96 | 2.37 | 0.90 |
| IL_16 | 18 | 5.32 | 9.71 | 0.11 | 1.76 | 38.43 | 0.01 |
| CD40L | 18 | 4.88 | 11.73 | 0.00 | 1.00 | 50.01 | 0.13 |
| VEGF_A | 18 | 5.49 | 13.89 | 0.00 | 0.84 | 49.58 | 0.75 |
| TSLP | 18 | 1.03 | 0.90 | 0.00 | 1.00 | 3.40 | 0.84 |
| IL_20 | 18 | 2.81 | 3.99 | 0.00 | 1.58 | 16.14 | 0.03 |
| ENA_78_CXCL5 | 18 | 1.03 | 0.64 | 0.02 | 0.99 | 2.40 | 0.99 |
| CD30 | 18 | 0.87 | 0.44 | 0.01 | 0.96 | 1.47 | 0.26 |
| TNF_RII | 18 | 1.98 | 3.24 | 0.00 | 0.97 | 14.29 | 0.32 |
| MDC | 18 | 9.98 | 31.76 | 0.00 | 0.99 | 136.13 | 0.82 |
| APRIL | 19 | 25.87 | 73.65 | 0.00 | 0.83 | 252.16 | 0.89 |
| TWEAK | 18 | 1.56 | 1.77 | 0.00 | 1.14 | 7.82 | 0.37 |

Statistical analysis based on Wilcoxon signed-rank tests and compares the fold-change with one.

**Supplemental Table 4.** **Fold change in biomarkers from pre-treatment to 4-8 weeks after initial vaccine dose for stage IV patients.**

| Biomarker | N | Mean | Std | Min | Median | Max | P-value |
| --- | --- | --- | --- | --- | --- | --- | --- |
| MCP_2_CCL8 | 35 | 2.44 | 7.82 | 0.00 | 0.86 | 46.36 | 0.28 |
| IL_2R | 35 | 15.91 | 72.69 | 0.00 | 0.86 | 422.15 | 0.49 |
| MIP_1_alpha_CCL3 | 35 | 2.08 | 2.98 | 0.00 | 0.99 | 12.86 | 0.55 |
| SDF_1_alpha | 35 | 737.84 | 3322.0 | 0.00 | 1.07 | 19436 | 0.19 |
| LIF | 36 | 1.36 | 1.79 | 0.00 | 0.89 | 10.77 | 0.85 |
| IP_10_CXCL10 | 35 | 2.05 | 3.60 | 0.00 | 1.07 | 17.77 | 0.14 |
| BLC_CXCL13 | 36 | 13.66 | 46.37 | 0.00 | 1.00 | 248.14 | 0.94 |
| Eotaxin_2_CCL24 | 35 | 25.06 | 96.49 | 0.00 | 0.70 | 445.56 | 0.18 |
| Eotaxin_CCL11 | 35 | 4.70 | 15.19 | 0.00 | 0.93 | 82.86 | 0.86 |
| IL_17A_CTLA_8 | 35 | 1.17 | 2.05 | 0.17 | 0.85 | 12.74 | 0.02 |
| SCF | 35 | 1.08 | 0.96 | 0.00 | 0.93 | 4.81 | 0.33 |
| G_CSF_CSF_3 | 35 | 2.15 | 4.09 | 0.00 | 1.00 | 20.89 | 0.66 |
| HGF | 35 | 5.98 | 19.89 | 0.00 | 0.83 | 93.58 | 0.99 |
| MIP_1_beta_CCL4 | 35 | 3.27 | 7.70 | 0.00 | 0.98 | 35.78 | 0.48 |
| MCP_1_CCL2 | 36 | 8.17 | 28.62 | 0.00 | 1.00 | 166.65 | 0.58 |
| MIF | 35 | 18.19 | 65.64 | 0.00 | 1.00 | 365.38 | 0.33 |
| I_TAC_CXCL11 | 35 | 44.83 | 224.45 | 0.00 | 1.00 | 1331.1 | 0.21 |
| TRAIL | 35 | 9.23 | 37.24 | 0.00 | 1.00 | 219.52 | 0.16 |
| MMP_1 | 36 | 26.23 | 149.03 | 0.00 | 0.67 | 895.41 | 0.09 |
| IL_15 | 35 | 1.15 | 1.38 | 0.00 | 0.98 | 6.35 | 0.24 |
| IL_18 | 35 | 2.31 | 6.64 | 0.00 | 0.86 | 37.12 | 0.07 |
| IL_16 | 35 | 18.90 | 78.17 | 0.05 | 1.02 | 451.64 | 0.48 |
| CD40L | 36 | 4.52 | 18.19 | 0.00 | 0.95 | 107.95 | 0.20 |
| VEGF_A | 36 | 5.70 | 21.56 | 0.00 | 0.76 | 126.67 | 0.29 |
| TSLP | 35 | 5.04 | 23.71 | 0.00 | 1.00 | 141.23 | 0.86 |
| IL_20 | 36 | 2.13 | 4.55 | 0.00 | 1.07 | 27.61 | 0.28 |
| ENA_78_CXCL5 | 35 | 13.78 | 61.86 | 0.00 | 0.78 | 355.39 | 0.01 |
| CD30 | 35 | 10.38 | 50.76 | 0.00 | 0.89 | 300.35 | 0.57 |
| TNF_RII | 36 | 7.01 | 21.26 | 0.00 | 0.97 | 100.21 | 0.84 |
| MDC | 35 | 7.33 | 24.04 | 0.00 | 0.94 | 129.44 | 0.81 |
| APRIL | 35 | 21.01 | 114.32 | 0.00 | 0.97 | 677.80 | 0.94 |
| TWEAK | 36 | 12.84 | 49.67 | 0.17 | 0.91 | 247.79 | 0.62 |

Statistically significant median decrease in CTLA-8 and CXCL-5 during the first 4-8 weeks of vaccine therapy (red). The data also suggest median decreases in MMP-1 and IL-18 at the trend level (blue).
