# Supplementary material for: Phase II trial of vaccination with autologous, irradiated melanoma cells engineered by adenoviral mediated gene transfer to secrete granulocyte-macrophage colony stimulating factor in patients with stage III and IV melanoma
Source: Front Oncol. 2024 May 15;14:1395978. doi: 10.3389/fonc.2024.1395978 (PMC11133610; doi:10.3389/fonc.2024.1395978)
Supplement: Supplementary file 4 [file Table_3.docx]

**Supplemental Table 5 – Delayed-Type Hypersensitivity Reactions**

|  | N |
| --- | --- |
| **Reaction at injection site?** | 82 |
| No |  |
| Yes | 42 |
| **Erythema?** | 80 |
| No |  |
| Yes | 44 |
| **Induration?** | 95 |
| No |  |
| Yes | 29 |
| **Warmth?** | 20 |
| Missing |  |
| No | 100 |
| Yes | 4 |
| **Redness?** | 20 |
| Missing |  |
| No | 79 |
| Yes | 25 |
| **Pruritis?** | 20 |
| Missing |  |
| No | 92 |
| Yes | 12 |
| **Swelling?** | 20 |
| Missing |  |
| No | 99 |
| Yes | 5 |
