# Supplementary material for: Phase II trial of vaccination with autologous, irradiated melanoma cells engineered by adenoviral mediated gene transfer to secrete granulocyte-macrophage colony stimulating factor in patients with stage III and IV melanoma
Source: Front Oncol. 2024 May 15;14:1395978. doi: 10.3389/fonc.2024.1395978 (PMC11133610; doi:10.3389/fonc.2024.1395978)
Supplement: Supplementary file 5 [file Table_4.docx]

**Supplemental Table 6**

1. **Stage IV Luminex Summary (Pretreatment)**

|  | | N | Mean | Std | Min | Median | Max |
| --- | --- | --- | --- | --- | --- | --- | --- |
| MCP_2_CCL8 | All | 34 | 172.0 | 676.1 | 0.0 | 8.5 | 3400.0 |
|  | Reaction | 23 | 249.0 | 816.4 | 0.0 | 7.3 | 3400.0 |
|  | Neither Erythema nor Induration |  |  |  |  |  |  |
|  | Erythema or Induration | 11 | 11.1 | 7.6 | 0.2 | 11.4 | 24.2 |
| IL_2R | All | 34 | 3562.3 | 5191.8 | 0.0 | 1833.2 | 28152 |
|  | Reaction | 23 | 3625.3 | 5815.0 | 0.0 | 2029.8 | 28152 |
|  | Neither Erythema nor Induration |  |  |  |  |  |  |
|  | Erythema or Induration | 11 | 3430.4 | 3812.3 | 0.0 | 1636.6 | 12170 |
| MIP_1_alpha_CCL3 | All | 34 | 59.4 | 111.7 | 0.0 | 27.5 | 625.4 |
|  | Reaction | 23 | 67.9 | 132.0 | 0.0 | 24.6 | 625.4 |
|  | Neither Erythema nor Induration |  |  |  |  |  |  |
|  | Erythema or Induration | 11 | 41.5 | 47.7 | 0.0 | 38.5 | 160.0 |
| SDF_1_alpha | All | 34 | 2038.3 | 2072.5 | 0.0 | 1528.6 | 5848.6 |
|  | Reaction | 23 | 1994.4 | 2146.3 | 0.0 | 1458.9 | 5848.6 |
|  | Neither Erythema nor Induration |  |  |  |  |  |  |
|  | Erythema or Induration | 11 | 2129.9 | 2006.3 | 0.0 | 2257.4 | 5769.4 |
| LIF | All | 34 | 74.4 | 239.4 | 0.0 | 7.0 | 1321.9 |
|  | Reaction | 23 | 102.6 | 288.5 | 0.0 | 8.3 | 1321.9 |
|  | Neither Erythema nor Induration |  |  |  |  |  |  |
|  | Erythema or Induration | 11 | 15.4 | 19.2 | 0.2 | 6.1 | 56.9 |
| IP_10_CXCL10 | All | 34 | 37.4 | 41.7 | 0.0 | 19.4 | 156.2 |
|  | Reaction | 23 | 39.4 | 46.6 | 0.0 | 20.0 | 156.2 |
|  | Neither Erythema nor Induration |  |  |  |  |  |  |
|  | Erythema or Induration | 11 | 33.1 | 30.5 | 0.0 | 16.9 | 97.0 |
| BLC_CXCL13 | All | 34 | 281.8 | 678.8 | 0.0 | 127.1 | 3862.0 |
|  | Reaction | 23 | 361.8 | 815.1 | 0.0 | 130.7 | 3862.0 |
|  | Neither Erythema nor Induration |  |  |  |  |  |  |
|  | Erythema or Induration | 11 | 114.6 | 114.4 | 0.0 | 123.6 | 359.9 |
| Eotaxin_2_CCL24 | All | 34 | 617.8 | 2828.5 | 0.0 | 83.3 | 16600 |
|  | Reaction | 23 | 844.0 | 3438.9 | 0.0 | 98.5 | 16600 |
|  | Neither Erythema nor Induration |  |  |  |  |  |  |
|  | Erythema or Induration | 11 | 145.0 | 147.1 | 1.0 | 57.0 | 368.3 |
| Eotaxin_CCL11 | All | 34 | 45.2 | 39.0 | 0.0 | 43.4 | 146.4 |
|  | Reaction | 23 | 45.9 | 42.9 | 0.0 | 28.6 | 146.4 |
|  | Neither Erythema nor Induration |  |  |  |  |  |  |
|  | Erythema or Induration | 11 | 43.6 | 31.3 | 0.4 | 47.7 | 96.3 |
| IL_17A_CTLA_8 | All | 34 | 538.8 | 1468.2 | 6.4 | 90.5 | 7121.5 |
|  | Reaction | 23 | 737.4 | 1759.8 | 6.4 | 97.9 | 7121.5 |
|  | Neither Erythema nor Induration |  |  |  |  |  |  |
|  | Erythema or Induration | 11 | 123.5 | 140.0 | 10.4 | 78.3 | 516.3 |
| SCF | All | 34 | 22.5 | 49.5 | 0.6 | 9.7 | 268.3 |
|  | Reaction | 23 | 28.3 | 59.4 | 0.6 | 9.7 | 268.3 |
|  | Neither Erythema nor Induration |  |  |  |  |  |  |
|  | Erythema or Induration | 11 | 10.3 | 8.0 | 0.6 | 9.8 | 25.7 |
| G_CSF_CSF_3 | All | 34 | 16.6 | 35.2 | 0.0 | 5.3 | 194.6 |
|  | Reaction | 23 | 18.4 | 41.3 | 0.0 | 4.4 | 194.6 |
|  | Neither Erythema nor Induration |  |  |  |  |  |  |
|  | Erythema or Induration | 11 | 12.9 | 17.9 | 0.0 | 9.2 | 62.7 |
| HGF | All | 34 | 100.6 | 136.9 | 0.6 | 64.8 | 655.1 |
|  | Reaction | 23 | 110.0 | 158.2 | 0.6 | 52.5 | 655.1 |
|  | Neither Erythema nor Induration |  |  |  |  |  |  |
|  | Erythema or Induration | 11 | 80.9 | 78.5 | 0.7 | 66.2 | 296.8 |
| MIP_1_beta_CCL4 | All | 34 | 15.8 | 13.7 | 0.6 | 15.1 | 49.3 |
|  | Reaction | 23 | 15.6 | 14.6 | 0.6 | 14.6 | 49.3 |
|  | Neither Erythema nor Induration |  |  |  |  |  |  |
|  | Erythema or Induration | 11 | 16.1 | 12.2 | 0.8 | 16.6 | 35.7 |
| MCP_1_CCL2 | All | 34 | 182.0 | 189.6 | 0.0 | 150.2 | 739.7 |
|  | Reaction | 23 | 149.6 | 168.9 | 0.0 | 119.0 | 634.7 |
|  | Neither Erythema nor Induration |  |  |  |  |  |  |
|  | Erythema or Induration | 11 | 249.8 | 220.1 | 0.9 | 233.7 | 739.7 |
| MIF | All | 34 | 331.4 | 787.2 | 0.0 | 26.8 | 2450.0 |
|  | Reaction | 23 | 362.2 | 828.5 | 0.0 | 29.7 | 2450.0 |
|  | Neither Erythema nor Induration |  |  |  |  |  |  |
|  | Erythema or Induration | 11 | 267.1 | 726.7 | 0.0 | 14.0 | 2450.0 |
| I_TAC_CXCL11 | All | 34 | 323.3 | 439.6 | 0.0 | 195.5 | 2215.5 |
|  | Reaction | 23 | 279.5 | 477.8 | 0.0 | 133.5 | 2215.5 |
|  | Neither Erythema nor Induration |  |  |  |  |  |  |
|  | Erythema or Induration | 11 | 414.9 | 349.0 | 2.4 | 270.8 | 911.1 |
| TRAIL | All | 34 | 143.9 | 283.6 | 0.0 | 31.8 | 1162.7 |
|  | Reaction | 23 | 157.7 | 310.2 | 0.0 | 27.8 | 1162.7 |
|  | Neither Erythema nor Induration |  |  |  |  |  |  |
|  | Erythema or Induration | 11 | 115.1 | 229.1 | 0.0 | 35.9 | 777.2 |
| MMP_1 | All | 34 | 2360.8 | 6365.4 | 0.0 | 121.1 | 31124 |
|  | Reaction | 23 | 1724.3 | 4558.7 | 0.0 | 115.6 | 21091 |
|  | Neither Erythema nor Induration |  |  |  |  |  |  |
|  | Erythema or Induration | 11 | 3691.6 | 9225.5 | 1.2 | 210.3 | 31124 |
| IL_15 | All | 34 | 46.6 | 111.0 | 0.0 | 9.7 | 536.0 |
|  | Reaction | 23 | 60.9 | 132.4 | 0.0 | 9.5 | 536.0 |
|  | Neither Erythema nor Induration |  |  |  |  |  |  |
|  | Erythema or Induration | 11 | 16.6 | 25.4 | 1.0 | 9.9 | 92.0 |
| IL_18 | All | 34 | 55.4 | 73.2 | 0.0 | 40.5 | 434.4 |
|  | Reaction | 23 | 62.4 | 86.9 | 1.8 | 39.6 | 434.4 |
|  | Neither Erythema nor Induration |  |  |  |  |  |  |
|  | Erythema or Induration | 11 | 40.9 | 26.9 | 0.0 | 41.4 | 86.0 |
| IL_16 | All | 34 | 473.1 | 618.5 | 0.0 | 256.2 | 3009.5 |
|  | Reaction | 23 | 539.9 | 695.0 | 0.0 | 291.9 | 3009.5 |
|  | Neither Erythema nor Induration |  |  |  |  |  |  |
|  | Erythema or Induration | 11 | 333.6 | 409.6 | 0.0 | 213.3 | 1208.4 |
| CD40L | All | 34 | 780.5 | 3255.4 | 0.0 | 81.4 | 19100 |
|  | Reaction | 23 | 1048.2 | 3952.0 | 0.0 | 70.8 | 19100 |
|  | Neither Erythema nor Induration |  |  |  |  |  |  |
|  | Erythema or Induration | 11 | 221.0 | 319.0 | 1.2 | 164.5 | 1043.6 |
| VEGF_A | All | 34 | 265.1 | 303.4 | 0.0 | 151.5 | 1222.1 |
|  | Reaction | 23 | 233.1 | 266.3 | 0.0 | 150.8 | 1122.0 |
|  | Neither Erythema nor Induration |  |  |  |  |  |  |
|  | Erythema or Induration | 11 | 331.9 | 374.9 | 1.3 | 221.1 | 1222.1 |
| TSLP | All | 34 | 886.7 | 4708.0 | 0.0 | 5.9 | 27500 |
|  | Reaction | 23 | 1293.3 | 5719.3 | 0.0 | 5.7 | 27500 |
|  | Neither Erythema nor Induration |  |  |  |  |  |  |
|  | Erythema or Induration | 11 | 36.6 | 83.8 | 0.0 | 6.1 | 285.5 |
| IL_20 | All | 34 | 59.5 | 128.1 | 0.0 | 18.8 | 708.1 |
|  | Reaction | 23 | 68.0 | 152.6 | 0.6 | 16.7 | 708.1 |
|  | Neither Erythema nor Induration |  |  |  |  |  |  |
|  | Erythema or Induration | 11 | 41.8 | 49.0 | 0.0 | 32.5 | 144.2 |
| ENA_78_CXCL5 | All | 34 | 599.0 | 915.6 | 0.0 | 336.2 | 4543.1 |
|  | Reaction | 23 | 755.9 | 1081.4 | 0.9 | 476.0 | 4543.1 |
|  | Neither Erythema nor Induration |  |  |  |  |  |  |
|  | Erythema or Induration | 11 | 270.9 | 136.9 | 0.0 | 246.9 | 459.5 |
| CD30 | All | 34 | 1303.5 | 3519.5 | 0.0 | 373.7 | 20252 |
|  | Reaction | 23 | 1637.8 | 4245.9 | 0.0 | 355.0 | 20252 |
|  | Neither Erythema nor Induration |  |  |  |  |  |  |
|  | Erythema or Induration | 11 | 604.4 | 648.6 | 1.9 | 392.5 | 1880.0 |
| TNF_RII | All | 34 | 177.7 | 130.3 | 0.0 | 211.7 | 405.4 |
|  | Reaction | 23 | 169.1 | 131.2 | 0.0 | 181.0 | 405.4 |
|  | Neither Erythema nor Induration |  |  |  |  |  |  |
|  | Erythema or Induration | 11 | 195.8 | 132.8 | 0.9 | 220.6 | 364.3 |
| MDC | All | 34 | 130.6 | 122.6 | 0.0 | 110.4 | 561.9 |
|  | Reaction | 23 | 133.0 | 142.4 | 0.0 | 130.6 | 561.9 |
|  | Neither Erythema nor Induration |  |  |  |  |  |  |
|  | Erythema or Induration | 11 | 125.8 | 70.3 | 0.0 | 109.2 | 231.0 |
| APRIL | All | 34 | 658.8 | 586.4 | 0.0 | 626.8 | 2359.8 |
|  | Reaction | 23 | 677.4 | 575.4 | 0.0 | 728.0 | 2359.8 |
|  | Neither Erythema nor Induration |  |  |  |  |  |  |
|  | Erythema or Induration | 11 | 620.0 | 635.6 | 11.0 | 368.9 | 1813.9 |
| TWEAK | All | 34 | 2125.2 | 3776.9 | 5.5 | 1204.7 | 21595 |
|  | Reaction | 23 | 2486.1 | 4502.5 | 5.6 | 1194.4 | 21595 |
|  | Neither Erythema nor Induration |  |  |  |  |  |  |
|  | Erythema or Induration | 11 | 1370.5 | 1244.9 | 5.5 | 1266.7 | 4324.0 |

1. **Stage IV Luminex Summary (median of 4 weeks post-vaccine; range 3.9 to 8 weeks)**

|  | | N | Mean | Std | Min | Median | Max |
| --- | --- | --- | --- | --- | --- | --- | --- |
| MCP_2_CCL8 | All | 32 | 201.1 | 765.8 | 0.0 | 6.8 | 3400.0 |
|  | Reaction | 22 | 288.1 | 916.7 | 0.0 | 6.5 | 3400.0 |
|  | Neither Erythema nor Induration |  |  |  |  |  |  |
|  | Erythema or Induration | 10 | 9.6 | 8.0 | 0.0 | 8.0 | 25.4 |
| IL_2R | All | 32 | 2830.6 | 5404.3 | 0.0 | 913.4 | 30014 |
|  | Reaction | 22 | 3156.7 | 6383.8 | 0.0 | 913.4 | 30014 |
|  | Neither Erythema nor Induration |  |  |  |  |  |  |
|  | Erythema or Induration | 10 | 2113.3 | 2163.1 | 0.0 | 1024.1 | 6323.7 |
| MIP_1_alpha_CCL3 | All | 32 | 52.9 | 105.3 | 0.0 | 21.5 | 572.1 |
|  | Reaction | 22 | 60.3 | 123.1 | 0.0 | 24.9 | 572.1 |
|  | Neither Erythema nor Induration |  |  |  |  |  |  |
|  | Erythema or Induration | 10 | 36.6 | 48.7 | 0.0 | 19.4 | 158.4 |
| SDF_1_alpha | All | 32 | 2427.5 | 3702.9 | 0.0 | 1355.7 | 19436 |
|  | Reaction | 22 | 2643.3 | 4263.3 | 0.0 | 1447.2 | 19436 |
|  | Neither Erythema nor Induration |  |  |  |  |  |  |
|  | Erythema or Induration | 10 | 1952.8 | 2110.2 | 0.0 | 1135.7 | 5240.7 |
| LIF | All | 33 | 68.0 | 260.4 | 0.0 | 6.5 | 1460.3 |
|  | Reaction | 22 | 94.8 | 317.7 | 0.0 | 6.7 | 1460.3 |
|  | Neither Erythema nor Induration |  |  |  |  |  |  |
|  | Erythema or Induration | 11 | 14.2 | 18.3 | 0.0 | 6.1 | 57.1 |
| IP_10_CXCL10 | All | 32 | 34.9 | 48.0 | 0.0 | 19.0 | 242.1 |
|  | Reaction | 22 | 37.5 | 55.8 | 0.0 | 18.1 | 242.1 |
|  | Neither Erythema nor Induration |  |  |  |  |  |  |
|  | Erythema or Induration | 10 | 29.2 | 24.8 | 0.0 | 20.4 | 76.9 |
| BLC_CXCL13 | All | 33 | 265.1 | 770.3 | 0.0 | 113.9 | 4409.4 |
|  | Reaction | 22 | 343.4 | 938.4 | 0.0 | 103.4 | 4409.4 |
|  | Neither Erythema nor Induration |  |  |  |  |  |  |
|  | Erythema or Induration | 11 | 108.6 | 95.9 | 0.0 | 119.7 | 256.1 |
| Eotaxin_2_CCL24 | All | 32 | 633.4 | 2918.2 | 0.0 | 35.1 | 16600 |
|  | Reaction | 22 | 839.1 | 3522.1 | 0.0 | 35.1 | 16600 |
|  | Neither Erythema nor Induration |  |  |  |  |  |  |
|  | Erythema or Induration | 10 | 180.8 | 237.4 | 0.0 | 35.7 | 691.0 |
| Eotaxin_CCL11 | All | 32 | 38.0 | 31.6 | 0.0 | 31.6 | 117.6 |
|  | Reaction | 22 | 37.4 | 31.7 | 0.0 | 34.3 | 117.6 |
|  | Neither Erythema nor Induration |  |  |  |  |  |  |
|  | Erythema or Induration | 10 | 39.2 | 33.2 | 0.0 | 31.6 | 87.4 |
| IL_17A_CTLA_8 | All | 32 | 480.1 | 1562.8 | 12.9 | 72.5 | 7682.0 |
|  | Reaction | 22 | 658.3 | 1868.7 | 17.8 | 84.7 | 7682.0 |
|  | Neither Erythema nor Induration |  |  |  |  |  |  |
|  | Erythema or Induration | 10 | 88.1 | 127.1 | 12.9 | 55.4 | 440.3 |
| SCF | All | 32 | 18.8 | 48.2 | 0.0 | 6.8 | 264.2 |
|  | Reaction | 22 | 23.6 | 57.7 | 0.0 | 6.7 | 264.2 |
|  | Neither Erythema nor Induration |  |  |  |  |  |  |
|  | Erythema or Induration | 10 | 8.2 | 6.4 | 0.6 | 7.1 | 17.2 |
| G_CSF_CSF_3 | All | 32 | 15.7 | 33.2 | 0.0 | 6.7 | 181.2 |
|  | Reaction | 22 | 16.3 | 39.3 | 0.0 | 4.4 | 181.2 |
|  | Neither Erythema nor Induration |  |  |  |  |  |  |
|  | Erythema or Induration | 10 | 14.3 | 13.3 | 0.0 | 11.4 | 40.3 |
| HGF | All | 32 | 76.1 | 111.6 | 0.0 | 46.4 | 625.6 |
|  | Reaction | 22 | 80.7 | 129.1 | 0.0 | 45.5 | 625.6 |
|  | Neither Erythema nor Induration |  |  |  |  |  |  |
|  | Erythema or Induration | 10 | 66.0 | 61.8 | 0.0 | 58.1 | 215.1 |
| MIP_1_beta_CCL4 | All | 32 | 12.5 | 10.7 | 0.0 | 12.5 | 32.9 |
|  | Reaction | 22 | 12.6 | 11.3 | 0.0 | 12.3 | 32.9 |
|  | Neither Erythema nor Induration |  |  |  |  |  |  |
|  | Erythema or Induration | 10 | 12.2 | 9.9 | 0.0 | 13.6 | 29.4 |
| MCP_1_CCL2 | All | 33 | 173.6 | 244.4 | 0.0 | 68.3 | 1095.5 |
|  | Reaction | 22 | 114.7 | 131.6 | 0.0 | 68.1 | 519.1 |
|  | Neither Erythema nor Induration |  |  |  |  |  |  |
|  | Erythema or Induration | 11 | 291.2 | 363.2 | 0.0 | 68.3 | 1095.5 |
| MIF | All | 32 | 273.2 | 713.2 | 0.0 | 34.6 | 2450.0 |
|  | Reaction | 22 | 270.2 | 707.6 | 0.0 | 34.6 | 2450.0 |
|  | Neither Erythema nor Induration |  |  |  |  |  |  |
|  | Erythema or Induration | 10 | 279.9 | 764.0 | 0.0 | 31.3 | 2450.0 |
| I_TAC_CXCL11 | All | 32 | 255.0 | 322.0 | 0.0 | 102.3 | 1331.1 |
|  | Reaction | 22 | 255.4 | 339.0 | 0.0 | 102.3 | 1331.1 |
|  | Neither Erythema nor Induration |  |  |  |  |  |  |
|  | Erythema or Induration | 10 | 254.1 | 298.4 | 0.0 | 174.2 | 916.3 |
| TRAIL | All | 32 | 137.9 | 232.4 | 0.0 | 36.8 | 890.1 |
|  | Reaction | 22 | 157.9 | 261.5 | 0.0 | 27.4 | 890.1 |
|  | Neither Erythema nor Induration |  |  |  |  |  |  |
|  | Erythema or Induration | 10 | 93.7 | 152.9 | 0.0 | 43.1 | 493.1 |
| MMP_1 | All | 33 | 2571.0 | 11739 | 0.0 | 35.1 | 67606 |
|  | Reaction | 22 | 370.9 | 623.6 | 0.0 | 38.9 | 2254.0 |
|  | Neither Erythema nor Induration |  |  |  |  |  |  |
|  | Erythema or Induration | 11 | 6971.3 | 20204 | 0.0 | 35.1 | 67606 |
| IL_15 | All | 32 | 38.9 | 113.7 | 0.0 | 6.9 | 552.0 |
|  | Reaction | 22 | 49.4 | 135.7 | 0.0 | 6.9 | 552.0 |
|  | Neither Erythema nor Induration |  |  |  |  |  |  |
|  | Erythema or Induration | 10 | 16.0 | 26.4 | 0.0 | 7.9 | 90.0 |
| IL_18 | All | 32 | 43.3 | 53.3 | 0.0 | 35.1 | 290.9 |
|  | Reaction | 22 | 51.6 | 62.1 | 0.0 | 38.1 | 290.9 |
|  | Neither Erythema nor Induration |  |  |  |  |  |  |
|  | Erythema or Induration | 10 | 25.1 | 16.9 | 0.0 | 20.8 | 50.4 |
| IL_16 | All | 32 | 421.5 | 601.6 | 3.4 | 250.9 | 3135.5 |
|  | Reaction | 22 | 478.8 | 696.2 | 3.4 | 268.4 | 3135.5 |
|  | Neither Erythema nor Induration |  |  |  |  |  |  |
|  | Erythema or Induration | 10 | 295.5 | 299.6 | 3.4 | 248.6 | 933.5 |
| CD40L | All | 33 | 753.9 | 3308.9 | 0.0 | 71.1 | 19100 |
|  | Reaction | 22 | 1039.0 | 4048.2 | 0.0 | 55.4 | 19100 |
|  | Neither Erythema nor Induration |  |  |  |  |  |  |
|  | Erythema or Induration | 11 | 183.8 | 292.6 | 0.0 | 81.2 | 971.0 |
| VEGF_A | All | 33 | 166.5 | 213.7 | 0.0 | 99.4 | 1070.9 |
|  | Reaction | 22 | 192.0 | 247.4 | 0.0 | 110.2 | 1070.9 |
|  | Neither Erythema nor Induration |  |  |  |  |  |  |
|  | Erythema or Induration | 11 | 115.4 | 115.5 | 0.0 | 74.2 | 347.5 |
| TSLP | All | 32 | 913.6 | 4855.5 | 0.0 | 4.4 | 27500 |
|  | Reaction | 22 | 1315.3 | 5853.2 | 0.0 | 1.7 | 27500 |
|  | Neither Erythema nor Induration |  |  |  |  |  |  |
|  | Erythema or Induration | 10 | 29.7 | 57.3 | 0.0 | 8.3 | 187.8 |
| IL_20 | All | 33 | 58.1 | 130.9 | 0.0 | 16.7 | 705.3 |
|  | Reaction | 22 | 63.0 | 153.1 | 0.7 | 15.9 | 705.3 |
|  | Neither Erythema nor Induration |  |  |  |  |  |  |
|  | Erythema or Induration | 11 | 48.5 | 73.7 | 0.0 | 20.9 | 248.5 |
| ENA_78_CXCL5 | All | 32 | 566.5 | 1271.7 | 0.0 | 211.0 | 7109.3 |
|  | Reaction | 22 | 742.6 | 1509.5 | 0.0 | 270.4 | 7109.3 |
|  | Neither Erythema nor Induration |  |  |  |  |  |  |
|  | Erythema or Induration | 10 | 179.2 | 109.5 | 91.9 | 145.9 | 380.8 |
| CD30 | All | 32 | 896.9 | 2090.7 | 0.0 | 309.0 | 10775 |
|  | Reaction | 22 | 1125.9 | 2492.0 | 0.0 | 309.0 | 10775 |
|  | Neither Erythema nor Induration |  |  |  |  |  |  |
|  | Erythema or Induration | 10 | 393.0 | 394.0 | 0.0 | 298.0 | 1231.9 |
| TNF_RII | All | 33 | 160.2 | 122.6 | 0.0 | 125.1 | 373.7 |
|  | Reaction | 22 | 150.1 | 120.2 | 0.0 | 124.9 | 373.7 |
|  | Neither Erythema nor Induration |  |  |  |  |  |  |
|  | Erythema or Induration | 11 | 180.4 | 130.6 | 2.4 | 203.8 | 358.8 |
| MDC | All | 32 | 114.3 | 106.1 | 0.0 | 99.6 | 471.2 |
|  | Reaction | 22 | 123.2 | 119.8 | 0.0 | 99.6 | 471.2 |
|  | Neither Erythema nor Induration |  |  |  |  |  |  |
|  | Erythema or Induration | 10 | 94.7 | 68.4 | 0.0 | 93.5 | 200.6 |
| APRIL | All | 32 | 630.5 | 704.4 | 0.0 | 491.0 | 3287.9 |
|  | Reaction | 22 | 657.3 | 782.8 | 0.0 | 491.0 | 3287.9 |
|  | Neither Erythema nor Induration |  |  |  |  |  |  |
|  | Erythema or Induration | 10 | 571.4 | 522.9 | 0.0 | 490.4 | 1353.1 |
| TWEAK | All | 33 | 1977.4 | 4003.8 | 60.3 | 951.0 | 22870 |
|  | Reaction | 22 | 2360.5 | 4830.4 | 122.9 | 969.7 | 22870 |
|  | Neither Erythema nor Induration |  |  |  |  |  |  |
|  | Erythema or Induration | 11 | 1211.2 | 1153.8 | 60.3 | 951.0 | 4037.5 |

1. Stage IV cohort comparing pretreatment Luminex data for patients who had a DTH reaction to those that did not (11 with reaction, 23 without). Comparisons between the two reaction groups were made using Wilcoxon-rank sum tests. For Stage IV disease, there were no statistically significant differences noted; however, differences in I_TAC_CXCL11 and ENA_78_CXCL5 were noted at the trend level (p=0.10 and 0.08, respectively). Levels of I_TAC_CXCL11 were higher in patients with induration/erythema; levels of ENA_78_CXCL5 were lower.
2. Luminex comparison conducted on samples that were collected a median of 4 weeks after first vaccine (range: 3.9 to 8 weeks). There were 50 patients with samples (32 Stage IV (10 with reaction)). For Stage IV, there were no comparisons that achieved statistical significance; however, the data suggest that IL 17A CTLA8 was lower in patients with reaction (p=0.08).
